# 

**Published:** 1884-12

**Authors:** 


					[i-\ -y^U "
| DECEMBER, 1884.
Vol. IIJ [No. 6.
THE
(^BRISTOL
INDEX?;
MEDICO-CHIRURGlCAt
JOURNAL
21 (SUiarterlg journal of tbe /nbeOical Sciences for tbe West
of SnglanD anD Soutb Males
PUBLISHED UNDER THE AUSPICES OF
THE BRISTOL MEDICO-CHIRURGICAL SOCIETY.
EDITED BY
J. GREIG SMITH,
M.A., F.R.S.E.
"Scire est nescire, nisi i6 rnc
Scirc alius scirct."
BRISTOL: J. W. ARROWSMITH ;
London : J. & A. Churchill, 11 New Burlington Street.
Price One Shilling and Sixpence.

				

